# Editorial: Cranial Placodes and Neural Crest Interactions in Craniofacial Development

**DOI:** 10.3389/fphys.2021.681397

**Published:** 2021-04-22

**Authors:** Jean-Pierre Saint-Jeannet, Patrick Blader, Lisa A. Taneyhill

**Affiliations:** ^1^Department of Molecular Pathobiology, College of Dentistry, New York University, New York, NY, United States; ^2^Unité de Biologie Moleculaire, Cellulaire et du Développement (MCD, UMR5077), Centre de Biologie Intégrative (CBI, FR 3743), Université de Toulouse, CNRS, UPS, Toulouse, France; ^3^Department of Animal and Avian Sciences, University of Maryland, College Park, MD, United States

**Keywords:** neural crest, placodes, craniofacial, vertebrates, sensory ganglia, skeleton

The vertebrate head is characterized by a complex craniofacial skeleton and paired sensory organs. These structures are derived from two adjacent embryonic cell populations, the neural crest and cranial placodes. The neural crest contributes to the craniofacial skeleton and a subset of cranial ganglia, while cranial placodes form the anterior pituitary, optic lens, inner ear, olfactory epithelium and several cranial ganglia. Defects in cranial neural crest and placode development can cause a wide array of human congenital malformations ranging from craniofacial disorders to hormone imbalance and sensory deficits. Throughout head development, reciprocal interactions between neural crest and placode cells are essential to drive the coordinated morphogenesis of multiple craniofacial structures. For this Research Topic, we have collected 14 research articles and reviews, analyzing and discussing at the cellular, molecular and genetic levels the role of the neural crest and cranial placodes in craniofacial development, and the importance of their interactions to organize the orofacial complex.

Neural crest and placode cells are truly evolutionary marvels. Together, they build much of the peripheral nervous system and sense organs that reside in the vertebrate head. Notably, the intricate interactions that occur between these two cell types endow vertebrates with features that distinguish them from their invertebrate ancestors. Indeed, comparative studies between jawless and jawed vertebrates have revealed molecular signatures of neural crest and placode cells, shedding light on the gene products and developmental signaling pathways that are involved in mediating neural crest-placode cell interactions in these distinct vertebrate lineages. York et al. discusses the origin of, and associations between, neural crest and placode cells in the jawed vertebrate head. Here, the authors delineate the gene regulatory networks (GRNs) that control neural crest and placode cell development and highlight how the coordinated movement and coalescence of neural crest and placode cells during embryogenesis is essential to yield functional sensory structures such as the cranial ganglia. These findings are reviewed in the context of jawed and jawless vertebrates, the latter relying on seminal results obtained from lamprey and hagfish. Importantly, recent studies demonstrate both evolutionary conservation and differences between jawed and jawless vertebrates with respect to the assembly of the cranial ganglia from neural crest and placode cells, generating a testable model for how these critical cell types appeared and worked in concert in ancestral vertebrates to pattern the craniofacial apparatus.

At the end of gastrulation, neural crest and cranial placodes arise from a narrow domain of the embryonic ectoderm immediately adjacent to the prospective neural plate, the neural plate border. In a pair of reviews, Thawani and Groves and Seal and Monsoro-Burq describe the major signaling molecules and transcription factors controlling the inductive and patterning events that elicit the development of these lineages at the neural plate border. Despite a growing understanding of the early GRN that controls the formation of the neural plate border during early embryonic stages, both reviews highlight important gaps in knowledge that will need to be resolved to understand how these two cell populations are established given their close proximity and similar developmental timeframe. The authors also consider outstanding questions in the field and how recent advances in transcriptomic analyses may help address these unresolved issues.

As discussed by Thawani and Groves and Seal and Monsoro-Burq, the neural plate border is defined by a unique signature of transcription factors among which Sox proteins are key players. Mutations in genes encoding SOX proteins have been linked to pathologies, often affecting multiple organ systems including the orofacial complex. Schock and LaBonne summarize the major classes of Sox factors and their role in the regulation of various aspects of neural crest development, including specification, multipotency retention, migration and differentiation. They highlight the importance of a subset of these genes in directing the development of neural crest- derived craniofacial structures through their unique ability to regulate craniofacial bone and cartilage formation, and drive palatogenesis, odontogenesis and salivary gland development. Finally they describe the clinical and molecular features of several SOXopathies, a group of rare multisystem developmental disorders. The prevalence of craniofacial defects in SOXopathies underscores the critical roles these factors have in the development and evolution of the vertebrate craniofacial complex.

In the last decade, modules of the neural crest GRN have been characterized in multiple organisms. This information has significantly improved our understanding of the mechanisms underlying the complex developmental trajectory of the neural crest. In their review, Chong-Morrison and Sauka-Spengler discuss recent advances in the characterization of the cranial neural crest GRN through state-of-the-art multi-omics approaches. They summarize how parallel considerations of transcriptome, interactome, and epigenome data sets have substantially refined the roles of the key players identified during the pre-omics era. They also discuss key questions that can now be addressed through a multi-omics approach and the development of new, unbiased functional genomics integration tools.

Neural crest cell migration is a process essential for the correct anatomical positioning of neural crest derivatives within the developing embryo. As highlighted in Barriga and Theveneau, neural crest cells employ a variety of different mechanisms, or “mixotaxis,” to ensure successful migration. Using *Xenopus* cranial neural crest cells as an example, the authors discuss different strategies that may be used by neural crest cells to accomplish migration. These include chemotaxis, in which cells respond to a gradient of soluble guidance factors, or haptotaxis, in which the cue is a bound signal; durotaxis, whereby cells move from softer to stiffer substrates; ratchetaxis, in which migratory cells are confined to a local route due to the presence of physical and/or chemical barriers; and galvanotaxis, whereby electric fields can influence cell migration. It is important to note that, with the preceding types of migratory mechanisms, it is often challenging to obtain *in vivo* data to support their existence due to the complex nature of the environmental milieu in the embryo, which directly impacts the migratory capacity of neural crest cells.

The molecules that mediate the formation, migration and differentiation of neural crest and placode cells have been the subject of investigation by many groups. In this Research Topic, Manohar et al. and Mo et al. tackle the former in the neural crest, identifying roles for Cadherin-11 and Platelet-Derived Growth Factor Receptors (PDGFRa and PDGRFb), respectively. While Cadherin-11 function in *Xenopus* neural crest cells (migration) and cancer cells (proliferation, survival, and migration) has been well-documented, studies by Manohar et al. in the chick embryo reveal that Cadherin-11 acts during neural crest cell specification. Cadherin-11 is first expressed in the neural plate, and is maintained throughout the formed neural tube and later observed in migratory neural crest cells. Knockdown of Cadherin-11 reduces the number of premigratory cranial neural crest cells and negatively impacts neural crest cell survival, in part, through activation of p53-mediated apoptosis. Ultimately this influences neural crest cell migration, as fewer neural crest cells are available to migrate, and those that do migrate do so poorly due to the absence of filopodia and lamellipodia. Collectively, these data point to multiple functions for Cadherin-11 in the forming neural crest.

Mo et al. investigated the role of receptor tyrosine kinase signaling in murine craniofacial development by ablating both *PDGFRa* and *PDGFRb* from the cranial neural crest. Through these studies, a genetic interaction could be identified between the two receptors and, notably, the presence of additional conditional alleles exacerbated phenotypes exhibited by the mutants. These phenotypes included increased distance between the nasal pits, facial hemorrhaging, misshapen fore- and/or midbrain, facial blebbing, and facial clefting. Further experiments revealed that PDGRa regulates the size and shape of the cranial neural crest cell migratory stream, with PDGFRb only contributing minimally. Both receptors, however, augment neural crest cell proliferation earlier in development, while during mid-gestation, PDGFRb signaling predominates with respect to controlling the proliferation of the craniofacial neural crest-derived mesenchyme. Interestingly, neither receptor functions during mid-gestation to control the survival of the cranial neural crest-derived mesenchyme. Taken together, these data reveal the roles of each PDGFR in the cranial neural crest and, subsequently, craniofacial development.

Canonical Wnt signaling is especially critical for neural plate border formation and craniofacial morphogenesis, and dysregulation of Wnt signaling has been linked to several craniofacial syndromes. To gain insights into these pathologies, Hutchins et al. sought to identify novel downstream targets of canonical Wnt signaling. To this end, they performed RNA-seq on sorted chick cranial neural crest cells overexpressing Draxin, a potent Wnt antagonist, which also interferes with the cranial neural crest epithelial-mesenchymal transition (EMT). The authors identified and validated a novel Wnt-responsive gene, RHOB, a member of the Rho GTP-binding protein family. Because RHOB is a known BMP-responsive gene in the trunk neural crest, by electroporation of a BMP reporter construct they confirmed the lack of active BMP signaling in cranial neural crest post-EMT, and demonstrated RHOB activation by expression of a stabilized form of b-catenin. These results highlight the importance of crosstalk between signaling pathways and axial level-specific interactions in the regulation of cranial neural crest development and craniofacial morphogenesis.

The complexity of the vertebrate head, containing as it does a wide variety of distinct neural crest and placodal derivatives, pleads for the importance of a multitude of specific molecular mechanisms in its development. In a genetic screen for regulators of craniofacial development, Dash et al. identified a mutant they named *snouty* that exhibits abnormalities of the facial prominences, cranial nerves and vasculature. Surprisingly, rather than affecting a gene involved specifically in craniofacial development, *snouty* codes for *Med23*, a subunit of the Mediator complex of proteins required for the transcriptional control of genes in all tissues. The allele isolated in the study represents a partial loss of *Med23* function that can be rescued, in part, by attenuating Wnt signaling. The results highlight the importance of undertaking forward genetic screens, even in systems such as the mouse. Furthermore, they reinforce the idea that so called housekeeping factors can have very specific developmental roles depending on the context.

Cranial neural crest cells express specific combinations of *Hox* genes based on their origin in the hindbrain. As they populate the pharyngeal arches (PA), neural crest cells respond to signals from the pharyngeal surface ectoderm and endoderm, which will determine their fate. Zhang et al. investigate the role of nested expression domains of *Hox* genes in the pharyngeal epithelium in the control of PA morphogenesis and differentiation. Transgenic mouse embryos with ectopic expression of Hoxb3 in the ectoderm pharyngeal epithelium of PA2 have multiple cranial nerve and skeletal defects. This phenotype correlates with a regionalized up-regulation of *Jag1* expression, a Notch ligand, in the ectoderm epithelium of PA2. The authors demonstrate through *in vivo* chromatin immunoprecipitation and *in vitro* luciferase reporter assays that Hoxb3 can directly bind to a cis-acting regulatory region at the *Jag1* locus to transactivate its expression. The work highlights the importance of pharyngeal epithelial and cranial neural crest cell interactions and the interplay between *Hox* genes and signaling molecules to promote PA and craniofacial development.

With the appearance of neural crest cells and placode cells in vertebrates, complex structures could be formed, such as the craniofacial skeleton, peripheral nerves, and paired sense organs. In his recent review, LaMantia posits that neural crest cells act as “inductive ambassadors” to promote the differentiation of craniofacial tissue and to serve as an initial conduit between the former and the brain. Neural crest-derived mesenchymal cells participate in interactions with epithelia and also directly supply signals to orchestrate tissue morphogenesis and differentiation. Using examples from normal embryogenesis (e.g., olfactory development) and various diseases possessing physical anomalies, cardiovascular defects and behavioral deficits (e.g., DiGeorge's Syndrome), data is put forth supporting the notion that the inductive cues provided by neural crest cells, along with neural crest-derived mesenchymal/epithelial interactions, are vital in driving tissue morphogenesis at sites outside of the face, such as the limbs and heart, and in forming proper neural circuits. Importantly, these studies have revealed signaling pathways and causative genes, the latter of which are expressed both in the brain and at sites of neural crest-derived mesenchymal/epithelial interactions in the developing embryo. Collectively, this review further underscores the intricate relationship and inherent cross-talk between the brain/neural tube, which initially possesses neural crest cells, and the face and other structures to which neural crest cells give rise.

The olfactory placodes form several cell types within the olfactory system such as gonadotropin releasing hormone-1 (GnRH-1) neurons, the first neuronal population present in the developing olfactory pit. However, the olfactory pit houses multiple neurons, including those expressing the transcription factor Islet1 (Isl1). To determine the role of Isl1 in GnRH development in the mouse, Taroc et al. document Isl1 expression, characterize the Isl1 lineage, and evaluate the phenotypes of GnRH neurons lacking Isl1. Isl1 is expressed in proliferating epithelial precursors that will give rise to neurons and later is noted in almost 90% of GnRH1-positive neurons in the olfactory pit. Lineage tracing of Isl1-expressing cells through the use of a tamoxifen inducible or constitutive *Cre* line results in the labeling of many different cell types. Finally, conditional knockout of *Isl1* in GnRH1 neurons revealed that Isl1 is not required for the migration of GnRH1 neurons nor expression of GnRH itself. In summary, these results raise the question as to the role of Isl1 in GnRH neuron development and point to the need for additional postnatal studies to shed light on Isl1 function.

Finally, Cheung et al. present the characterization of a rare cell type in the olfactory epithelium of the zebrafish. Olfactory rod cells display a prominent actin-rich projection at their apical surface. While not previously described in zebrafish, these cells have already been reported in other teleost but whether they represent artifacts of fixation has until now remained a possibility. In the present study, live imaging was used to verify the presence of an actin-rich rod unequivocally. Although the function of these rare cells remains a mystery, possibilities range from odor-sensing to a role in immunity, in a manner similar to brush or tuft cells described in mammals. Future studies will be needed to address these possibilities, and to determine the developmental origin of this intriguing cell type.

## Perspectives

As illustrated by these contributions, cranial placode and neural crest cells are engaged in reciprocal interactions throughout their developmental history that influence their fate, behavior and ultimate differentiation. While much has been learned in the past decade on the importance of these interactions during vertebrate head development, it remains a burgeoning field. With the introduction of multi-omic and single cell analyses, the next decade is expected to bring a wealth of new information on the development of these two cell types. One important challenge moving forward is to further refine the hierarchy of signaling molecules and downstream transcriptional regulators that form the basis of the placode and neural crest GRNs in order to understand how these adjacent cell populations are independently established at the neural plate border. It is especially critical to validate functionally the direct vs. indirect regulations among these factors. Another unresolved question relates to the molecular bases of placode and neural crest multipotency, a topic that is still under active debate. Through deployment of a wide array of signaling cues, placode and neural crest cells cross-regulate their respective migration and differentiation, but how these cells integrate inputs from multiple sources, both spatially and temporally, to build the craniofacial complex remains obscure and is essential to investigate. These are just a handful of questions and challenges that the craniofacial biology community will likely face in the upcoming years. We are extremely grateful to all the authors and reviewers who made this collection possible. We hope these articles will stimulate further interest and inspire young scientists to tackle some of these and other outstanding questions in the field.

## Author Contributions

All authors listed have made a substantial, direct and intellectual contribution to the work, and approved it for publication.

## Conflict of Interest

The authors declare that the research was conducted in the absence of any commercial or financial relationships that could be construed as a potential conflict of interest.

